# Comparison of Stress Distribution in Fixed Partial Dentures With an Endocrown Abutment Made of Two Ceramic Materials and Different Residual Tooth Structures, Using Finite Element Analysis

**DOI:** 10.1155/ijod/8823417

**Published:** 2026-02-10

**Authors:** Siavash Asadi Paein Lamooki, Simindokht Zarrati, Fariba Mahmoudi Yamchi, Nikfam Khoshkhounejad

**Affiliations:** ^1^ Department of Prosthodontics, School of Dentistry, Tehran University of Medical Sciences, Tehran, Iran, tums.ac.ir; ^2^ Dental Research Center, Dentistry Research Institute, School of Dentistry, Tehran University of Medical Sciences, Tehran, Iran, tums.ac.ir

**Keywords:** endocrown, finite element analysis, fixed partial denture, stress distribution

## Abstract

**Statement of Problem:**

The use of endocrowns as retainers for fixed partial dentures (FPD) may be considered questionable.

**Purpose:**

This study aimed to compare stress distribution in FPD with an endocrown abutment made of two ceramic materials and different residual tooth structures.

**Materials and Methods:**

Models with a missing mandibular right first premolar and an endodontically treated second premolar with two different residual tooth wall height (RWH) of 4.5 and 3 mm were designed in CAD software. These models received two types of FPD made of lithium disilicate and zirconia ceramics and were subjected to occlusal and buccal force conditions. Finally, the von Mises stress distribution was assessed using ANSYS software.

**Results:**

Comparison of stress distribution revealed that the model with 4.5 mm of RWH experienced a lower maximum von Mises stress than the model with 3 mm of RWH. Irrespective of load points, under both occlusal and buccal forces, the connector region is the area of greatest stress concentration. The results indicated that in all scenarios, the maximum von Mises stress in zirconia FPD was more than in lithium disilicate FPDs.

**Conclusion:**

Increasing the extent of tooth preparation and structural damage will lead to higher von Mises stress. The highest maximum von Mises stresses were generated in the zirconia models and buccal load conditions. Both materials and designs were acceptable and could be used as FPDs with an endocrown as a retainer.

**Clinical Implications:**

The use of endocrowns as retainers for FPD can be considered as an alternative in certain cases, offering a balance between conservation of tooth structure and mechanical stability.

## 1. Introduction

For reconstructing root‐treated teeth with structural destruction by the indirect restorative approach, two scenarios exist: (1) anchorage from the canals and post‐core construction, and (2) adhesive restorations without using anchorage inside the root canal. Recent advancements in dental bonding agents and composite resin cements have made it possible to create indirect restorations without the use of posts for retention. This structure is known as an “endocrown” and was introduced in 1999 by Bindle and Mormann, which was later modified by some researchers and clinicians [1]. The clinical success rate of single endocrowns has been reported in the range of 94% to 100% [2]. Considering various conditions, endocrowns have been recognized as a suitable treatment option in minimally invasive dentistry for a single tooth with a history of endodontic treatments, both in terms of restoration, esthetics, and clinical aspects [3].

Usually dental implant is the preferred treatment for replacing a missing tooth adjacent to intact teeth [4, 5]. However, clinical contraindications (such as smoking, uncontrolled diabetes, or multiple cancer types) or other conditions (such as economic difficulties or fear of surgery) can hinder the selection of the optimal treatment plan [5, 6]. In such cases, the traditional approach is to prepare adjacent teeth for full‐coverage restoration, as the retainers of an FPD. However, in this process, ~63% to 73% of the tooth structure must be removed [7]. In contrast, the endocrown method preserves more of the tooth, does not require gingival lengthening surgery and has less invasive nature [8 and 9]. Some studies have recommended FPDs with endocrown retainers as a proper alternative for conventional FPDs [10]. Yet, not enough research has been conducted on endocrowns as the retainer of fixed partial dentures (FPD).

Finite element analysis (FEA) is one of the recommended laboratory methods before clinical recommendation and implementation [11]. Therefore, by using FEA, von Mises stress levels in the entire model and its various components were assessed to evaluate endocrown as a retainer for FPD. Since the elastic modulus of the material and the remaining tooth structure may affect the stress distribution and fracture resistance of the complex, to figure out the best possible design for the FPD with an endocrown retainer, two different materials and two different remaining tooth structures were considered [12].

For assessing the potential use of endocrown as a retainer of FPD, this study was designed to compare stress distribution in FPD with an endocrown abutment made of two ceramic materials and different residual tooth structures.

## 2. Material and Methods

A dental model was scanned by a Ceramill Motion 2 scanner (Amann Girrbach AG) (scan 1). The mandibular right first premolar was then omitted. The mandibular right canine tooth received a 2 mm incisal surface reduction and a 1.5 mm reduction of axial surfaces with a radial shoulder finish line for a full‐coverage restoration (Figure 1). The mandibular right second premolar tooth was considered as an endodontically treated tooth and later prepared for endocrown restoration (Figure 1). For this purpose, the clinical crown of the tooth was reduced as a butt joint in such a way that 4.5 mm of clinical crown remained, which will be referred to as remaining wall height (RWH). The mesial wall was also reduced to 1 mm above the cementoenamel junction. Then, 2 mm of the peripheral tooth structure was preserved. Dental roots and pulp chamber were later designed by Mimics software (Materialise). After completion of tooth preparation, the two abutments were evaluated in terms of parallel path of insertion, and the possible undercuts in the path of insertion were removed. Next, the model was scanned again with the same scanner (scan 2). After scanning, the second premolar tooth was occlusally reduced again for another 1.5 mm. Accordingly, 3 mm of crown height remained. Another scan was then obtained (scan 3).

Figure 1Teeth preparations: (a) the mandibular right canine tooth preparation for a full‐coverage restoration and (b) the second premolar tooth preparation for an endocrown restoration.

**Figure:**
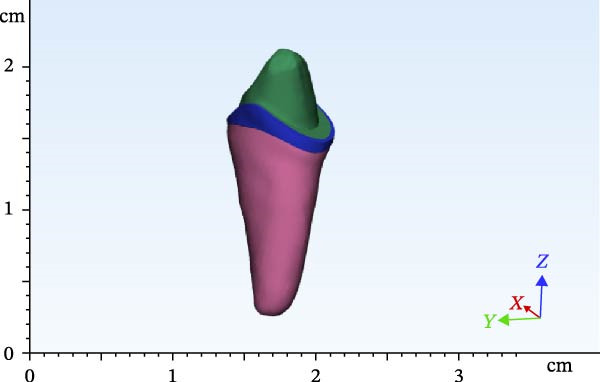
(a)

**Figure:**
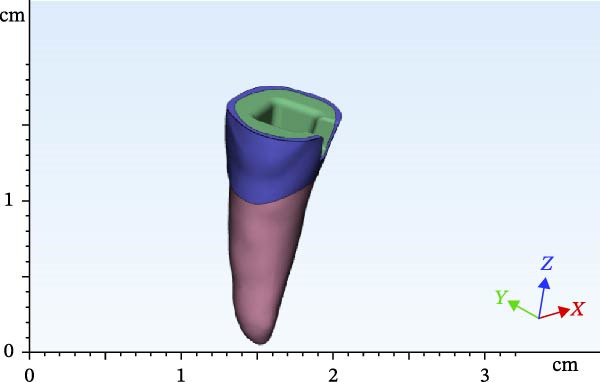
(b)

**Figure 3:**
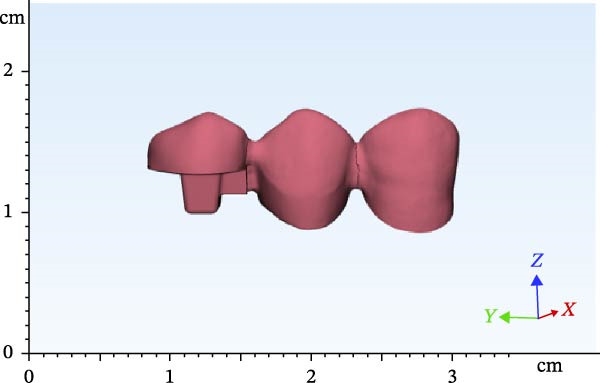
Designed FPD in the 4.5 RWH in premolar.

**Figure 4:**
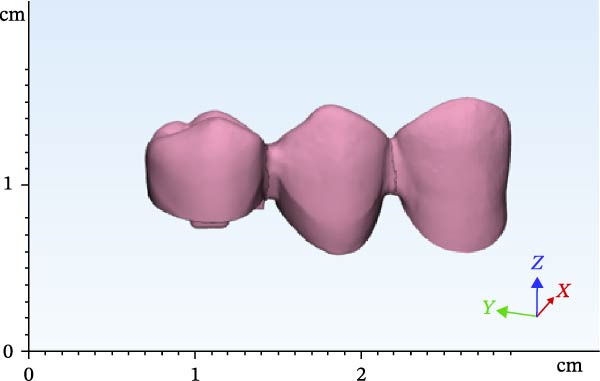
Designed FPD in the 3 RWH in premolar.

The scanned file was transferred to Mimics software. Alveolar bone was designed surrounding the roots, which included two parts of cortical and cancellous bone. The area of the canine and first and second premolars was selected, removed from the model, and replaced with scans 2 and 3 in two different files. Designing was then completed by adding the pulp space, root canal, and layering of enamel and dentin. Access cavity with 6‐degree taper of axial walls was designed. Finally, the second premolar was designed and modeled as an endodontically treated tooth with gutta‐percha present in the root canal. Subsequently, according to scan 1, FPDs were designed with two different materials with one endocrown abutment and one full‐coverage abutment (Figures 2–4). Multilink Automix resin cement (Ivoclar AG) with 0.12 mm cement thickness was selected and designed. The periodontal ligament (PDL) and trabecular and cortical bones were also reconstructed and modeled. The PDL had 0.2 mm thickness, and cortical bone and lamina dura had 2 mm and 0.3 mm thickness, respectively [13].

Figure 2Endocrowns designed for different RWHs: (a) 4.5 mm RHW and (b) 3 mm RHW.

**Figure:**
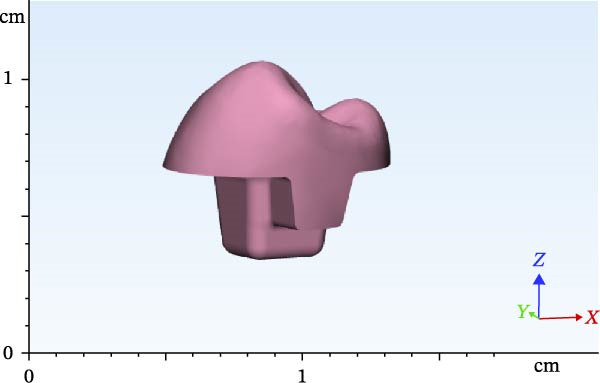
(a)

**Figure:**
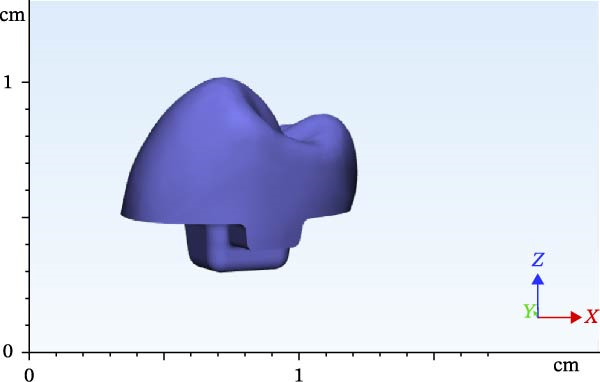
(b)

After completion of designing, all models in STL format were exported from Mimics and 3‐Matics to Geomagic software (3D Systems, Inc.) to be converted to CAD files in STP format (volumetric and three‐dimensional) for subsequent analysis in ANSYS Workbench 19.2 (ANSYS Inc., Houston, TX, USA). In ANSYS software, first the mechanical properties of all materials and related structures were recorded. Table 1 presents the Poisson’s ratio and elastic modulus of different components. Meshing was then performed. The total number of tetrahedral elements was 300,601 and the number of nodes was 495,946.

**Table 1:** Material properties.

Material	Elastic modulus (GPa)	Poisson ratio
Enamel [14]	84.10	0.33
Dentin [14]	18.60	0.31
Pulp [15]	0.0068	0.45
PDL [16]	0.07	0.45
Cortical bone [16]	17	0.30
Cancellous bone [16]	0.35	0.25
Lithium disilicate [17, 18]	100	0.20
Zirconia [19]	210	0.30
Multilink automix cement [20]	5	0.29
Gutta percha [15]	0.07	0.40

Numerical analyses were carried out using ANSYS software. Boundary conditions were applied, and the desired loading conditions were simulated. The pattern of distribution of von Mises stress values was then analyzed in the entire assembly and separately in each component, such as cement, residual wall, and prostheses.

### 2.1. Boundary Conditions

The bone was fixed, and the teeth preserved their natural micromovements in bone depending on the applied load and elasticity of the PDL. Also, all components were considered linearly elastic and homogeneously isotropic with tight bonds.

### 2.2. Load Application

**Figure 5:**
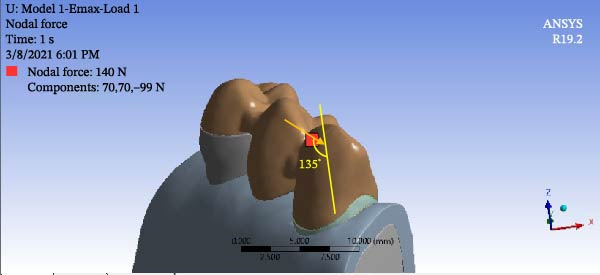
Force application in the first scenario for canine.

**Figure 6:**
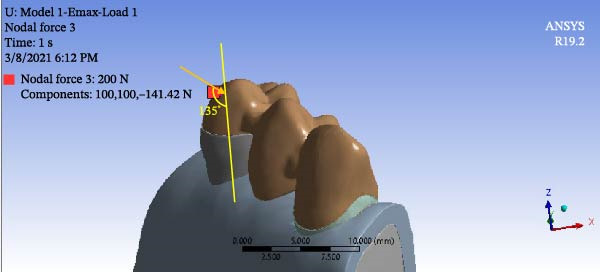
Force application in the first scenario for premolar teeth.

**Figure 7:**
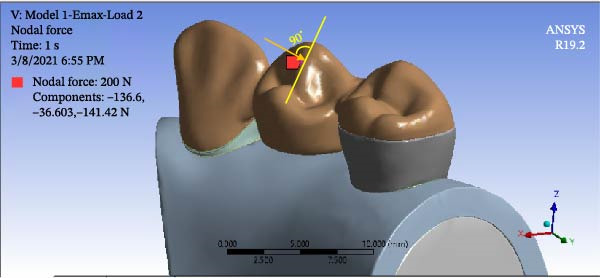
Force application in the second scenario.

To simulate group function occlusion for eccentric movement, loads were applied from the buccal surface at 1 mm distance from the cusp tip with a 135‐degree angle relative to the longitudinal axis of the tooth. The magnitude of load was 140 N for the canine (Figure 5) and 200 N for premolar teeth (Figure 6). To simulate centric contacts, in another scenario, a 200 N load was applied vertically to the lingual slope of the buccal cusp of premolar teeth at the interface of the buccal one‐third and lingual two‐thirds. No load was applied to the canine tooth in this mode (Figure 7) [21, 22].

## 3. Results

**Figure 9:**
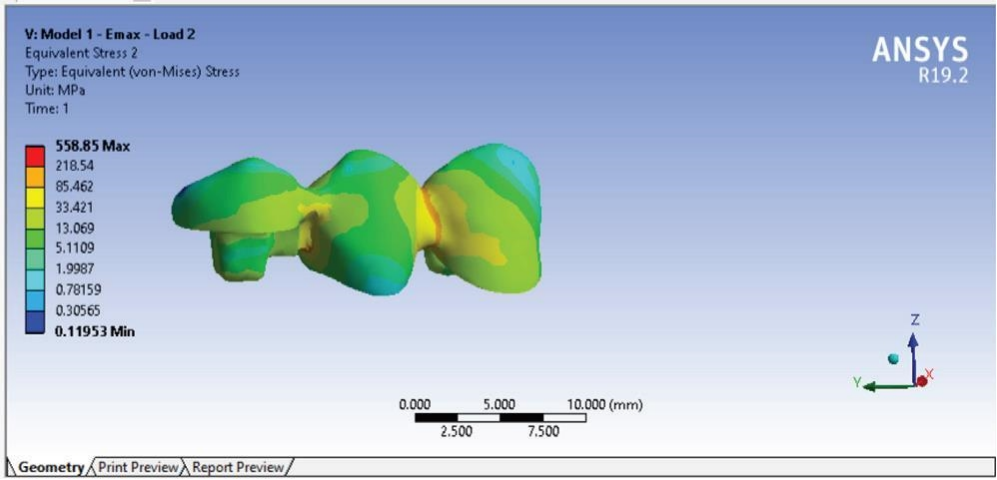
Stress distribution and maximum von Mises stress FPD under occlusal force.

**Figure 10:**
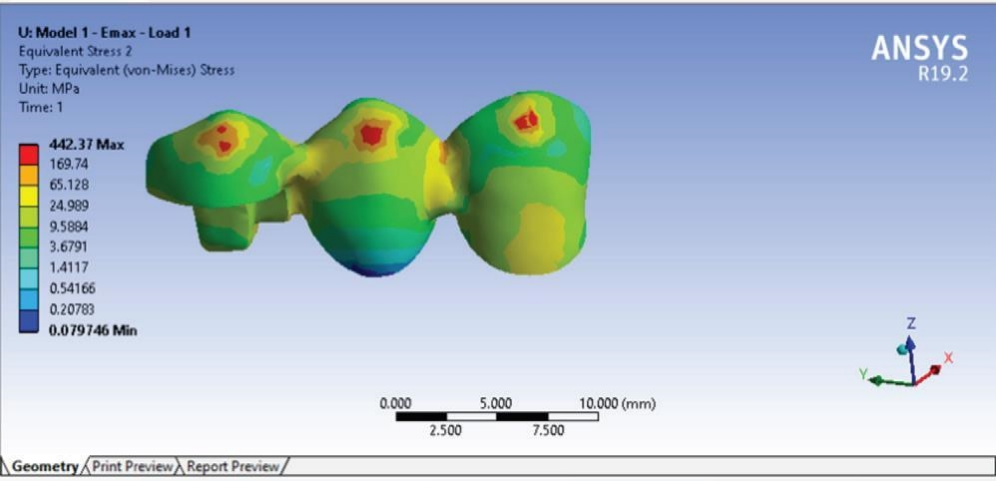
Stress distribution and maximum von Mises stress FPD under buccal force.

The results of maximum stress, generated in the FPDs of different material, in different RWH, and in different force directions, are summarized in Table 2. By comparing the stress distribution in the residual dental structure after preparation, it was determined that in the model where the RWH in the second premolar was 4.5 mm, the maximum von Mises stress generated was lower than in the model with 3 mm of RWH. However, the stress distribution patterns in both models were similar (Figure 8). The results of stress distribution analysis in the designed FPD showed that in both Emax and Zirconia materials, under both occlusal and buccal forces, not considering the load points, the connector regions tolerated the highest stress levels (Figures 9, 10).

Figure 8Stress distribution and maximum von Mises stress in model (a) 3 mm RWH (b) 4.5 mm RWH.

**Figure:**
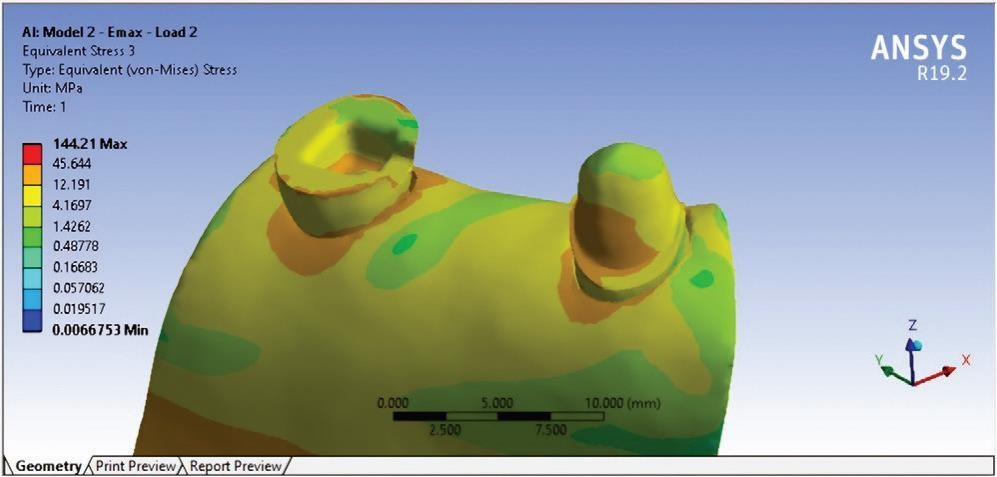
(a)

**Figure:**
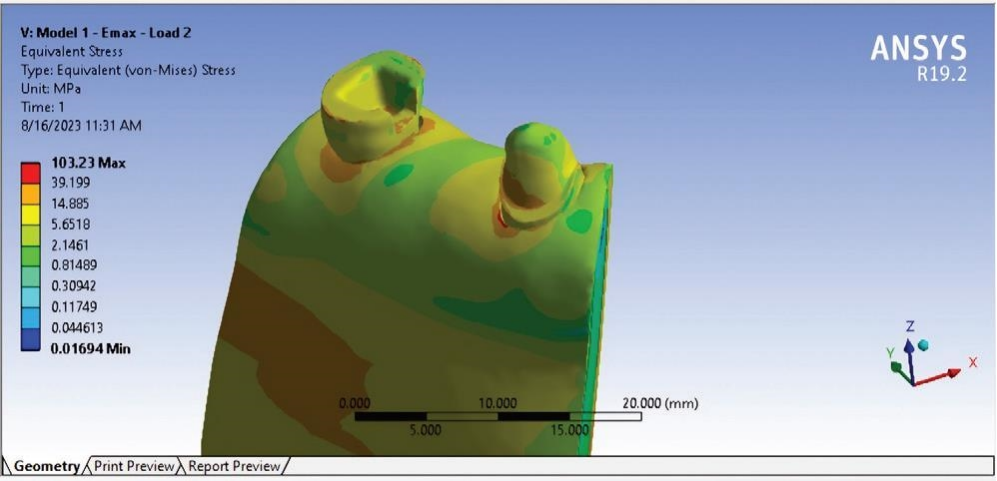
(b)

**Table 2:** Maximum stress in MPa, generated in different materials, in different RWH and different force direction.

Material	3 RWH		4.5 RWH	
	Buccal force	Occlusal force	Buccal force	Occlusal force
Zirconia	581.96	508.1	612.9	582.2
Lithium disilicate	388.2	378.1	402.37	358.85

The results showed that in all models, under both occlusal and buccal forces, the maximum von Mises stress in the zirconia FPD material was higher than that of the lithium disilicate material. Also, in all models, in either 3 or 4.5 of RWH, buccal forces generated higher stresses. All of the data obtained for maximum stress generated in both materials are below the ultimate compressive strength of the materials (~2000 MPa for zirconia and ~449 MPa for lithium disilicate) [23, 24]. Also, in none of the scenarios did the compressive stress in the dentin exceed its ultimate compressive strength.

## 4. Discussion

The present study, using FEA, compared stress distribution in FPDs with one endocrown and one full‐coverage abutment, fabricated from two ceramic types and with two different RWHs. Because of the limitations of lithium disilicate for posterior FPD, in the design process, FPDs on the mandibular canine and second premolar were considered. The results of the current study demonstrated that, when there is a higher residual wall in the tooth, the maximum von Mises stress generated is lower. The stress distribution patterns in both models were the same. Such findings can emphasize the importance of preserving the tooth structure, during the root canal therapy or endocrown preparation, as much as possible. The FEA study evaluated different endocrown heights with the same RWH and reported almost the same or lower stress by the increasing of restoration height [25]. Whereas in the current study’s design, considering a fixed occluso‐gingival height for the tooth, by increasing the restoration height, the RWH was decreased and vice versa, and it resulted that the restoration’s height is proportional to the stress amount. Therefore, the different results of the two studies can be justified.

In the present study, in agreement with da Fonseca et al. [25], it is demonstrated that oblique force increases the stresses in the complex. In the design and occlusal adjustment of restorations, care must be taken to align the forces more longitudinally, and engaging in lateral movements can jeopardize the restoration. The results of stress distribution analysis in the designed FPD showed that in both Emax and Zirconia materials, under both occlusal and buccal forces, the connector regions tolerated the highest stress levels. Also, other literature had referred to the concentration of stress in the connector regions of the FPDs and catastrophic failures rising from this part [10, 26]. Appropriate dimensions of the connector area are essential to prevent bending and fracture [26, 27]. Therefore, the design of FPDs’ connectors should restrictively follow the manufacturer’s instructions for the intended material, and the importance of correct laboratory workflow in this area should be highly emphasized. In the present study, the size of the connector was 16 mm^2^ in LDS FPD [28] and 9 mm^2^ for zirconia FPD as instructed by the manufacturers [29]. It should be noted that, despite the small proximal surface of the endocrown, which is limited to a box, it is the site of stress accumulation, and the standard surface area recommended by the manufacturer should not be compromised. In the design of the present study, the small occluso‐gingival surface of the connector of the endocrown restoration was compensated by extension of the connector in the bucco‐lingual direction until reaching the sufficient surface area.

In all models and under both types of occlusal and buccal forces, the maximum von Mises stress in the zirconia FPD was higher than that of the lithium disilicate FPD. This result demonstrated that when the elastic modulus of the FPD material increased, the maximum von Mises stress in FPD increased. This was in agreement with Zhu et al. [12]. The stresses were within the tolerable range for both materials. No fractures are expected, and both materials were acceptable for both designs. Therefore, they can be used as FPDs with the current design. Results of an in vitro study demonstrated that Katana zirconia exhibits the highest initial fracture strength in comparison with VITA Enamic, IPS e.max CAD, and BioHPP [30]. In the application of FEA, inherent limitations, such as considering linear elasticity coefficients and assuming homogeneity in structures, must be taken into account. Additionally, it should be noted that stress distribution patterns may vary depending on the material type, the properties of different layers in the models, and the models used in different experiments [31]. Therefore, the results of this study are not applicable to clinical settings until further clinical research is conducted. A future finite element study is suggested to compare the performance of FPDs with endocrown retainers with conventional FPDs retained by full‐coverage crowns.

## 5. Conclusion

1. The maximum von Mises stress generated in the model with a higher residual tooth wall in the premolar was lower.
2. The maximum von Mises stress generated in the zirconia models was more than in the lithium disilicate models.
3. Buccal loading generated more maximum von Mises stress than occlusal loading.

## Funding

The authors received no specific funding for this work.

## Conflicts of Interest

The authors declare no conflicts of interest.

## Data Availability

All data during this study are available from the corresponding author upon reasonable request.
